# Retracted: Human Interferon Inducible Transmembrane Protein 3 (IFITM3) Inhibits Influenza Virus A Replication and Inflammation by Interacting with ABHD16A

**DOI:** 10.1155/2022/9790782

**Published:** 2022-04-06

**Authors:** BioMed Research International

*BioMed Research International* has retracted the article titled “Human Interferon Inducible Transmembrane Protein 3 (IFITM3) Inhibits Influenza Virus A Replication and Inflammation by Interacting with ABHD16A” [1] due to concerns with the reliability of the data. We have been made aware of a number of concerns and inconsistencies with the data and results presented. Specifically, the images presented in Figure 2(b) do not correspond to the stains used and the blots in Figure 1, 3, and 4 do not correspond to the experiment described. The article was also found to contain a substantial amount of material from the two thesis articles [2, 3].

The authors contacted the journal to request the retraction of the article due to these concerns, explaining that the research was conducted by an external company. With the agreement of the authors and the editorial board the article is being retracted from the journal.
